# Practical applications of photon-counting CT in oncology: an organ-based pictorial review

**DOI:** 10.1186/s13244-026-02399-x

**Published:** 2026-10-09

**Authors:** Yusuke Kawasaki, Kota Yokoyama, Hiroto Hada, Hirofumi Yamada, Junichi Tsuchiya, Kurara Yamamoto, Iichiroh Onishi, Yosuke Ariizumi, Takayuki Honda, Kenichi Okubo, Tomoyuki Aruga, Daisuke Asano, Daisuke Ban, Soichiro Yoshida, Yasuhisa Fujii, Kimio Wakana, Marie Hanaoka, Keisuke Tanaka, Masahide Yamamoto, Takehiko Mori, Sadakatsu Ikeda, Ukihide Tateishi

**Affiliations:** 1 Department of Diagnostic Radiology and Nuclear Medicine, Institute of Science Tokyo, Tokyo, Japan; 2 Radiology Center, Institute of Science Tokyo Hospital, Tokyo, Japan; 3 Department of Pathology, Institute of Science Tokyo Hospital, Tokyo, Japan; 4 Department of Comprehensive Pathology, Graduate School of Medical and Dental Sciences, Institute of Science Tokyo, Tokyo, Japan; 5 Department of Head and Neck Surgery, Institute of Science Tokyo, Tokyo, Japan; 6 Department of Respiratory Medicine, Institute of Science Tokyo, Tokyo, Japan; 7 Department of Thoracic Surgery, Institute of Science Tokyo, Tokyo, Japan; 8 Department of Breast Surgery, Institute of Science Tokyo, Tokyo, Japan; 9 Department of Hepatobiliary Surgery, Institute of Science Tokyo, Tokyo, Japan; 10 Department of Urology, Institute of Science Tokyo, Tokyo, Japan; 11 Department of Obstetrics & Gynecology, Institute of Science Tokyo, Tokyo, Japan; 12 Department of Gastrointestinal Surgery, Institute of Science Tokyo, Tokyo, Japan; 13 Department of Hematology, Institute of Science Tokyo, Tokyo, Japan; 14 Department of Precision Medicine and Cancer, Institute of Science Tokyo, Tokyo, Japan

**Keywords:** Tomography (X-ray computed), Neoplasms, Diagnostic imaging, Contrast media, Iodine compounds

## Abstract

**Abstract:**

Spectral computed tomography (CT) has become an important extension of contrast-enhanced CT (CECT) in oncologic imaging by providing material-specific information beyond conventional morphologic assessment. Dual-energy CT (DECT), implemented on energy-integrating detector CT (EID-CT) platforms, has demonstrated the value of low-keV virtual monoenergetic imaging (VMI), iodine mapping, and material decomposition for improving tumor conspicuity, delineating local extent, and supporting tissue characterization. However, broader clinical adoption of EID-CT-based DECT has been limited by increased low-keV image noise and workflow complexity. Photon-counting detector CT (PCD-CT) addresses these limitations by offering improved energy resolution, reduced electronic noise, and routine acquisition of multi-energy data without dedicated dual-energy protocols. In particular, low-keV VMI around 40 keV can be generated with clinically acceptable noise, enabling consistent enhancement-based assessment in daily practice. Iodine-based reconstructions further complement morphologic evaluation by visualizing viable tumor enhancement and enabling quantitative analysis. This organ-based pictorial review summarizes practical applications of PCD-CT–based spectral imaging in oncology. Across organ systems, spectral CT may provide complementary problem-solving information by improving lesion detection, tumor extent delineation, and diagnostic confidence, particularly when conventional CT findings are equivocal, or MRI is unavailable, contraindicated, or limited. Emerging quantitative parameters, including electron density, effective atomic number, and advanced material decomposition, may further expand oncologic CT applications, although outcome-driven validation remains limited. Together, these features position PCD-CT as a practical spectral imaging platform for routine oncologic CT.

**Key Points:**

- **Question** How can PCD-CT address low-keV image noise and workflow barriers that have limited routine DECT-based spectral imaging in oncologic CT practice?
- **Findings** PCD-CT enables low-keV VMI and iodine-based reconstructions that may improve lesion conspicuity, tumor-extent assessment, and problem-solving across organ-specific oncologic workflows.
- **Critical relevance statement** This review clarifies practical, evidence-aware uses of PCD-CT spectral reconstructions in oncology, highlighting low-keV VMI as an adjunct, selective iodine-map problem-solving, and investigational quantitative parameters.

**Graphical Abstract:**

**Figure:**
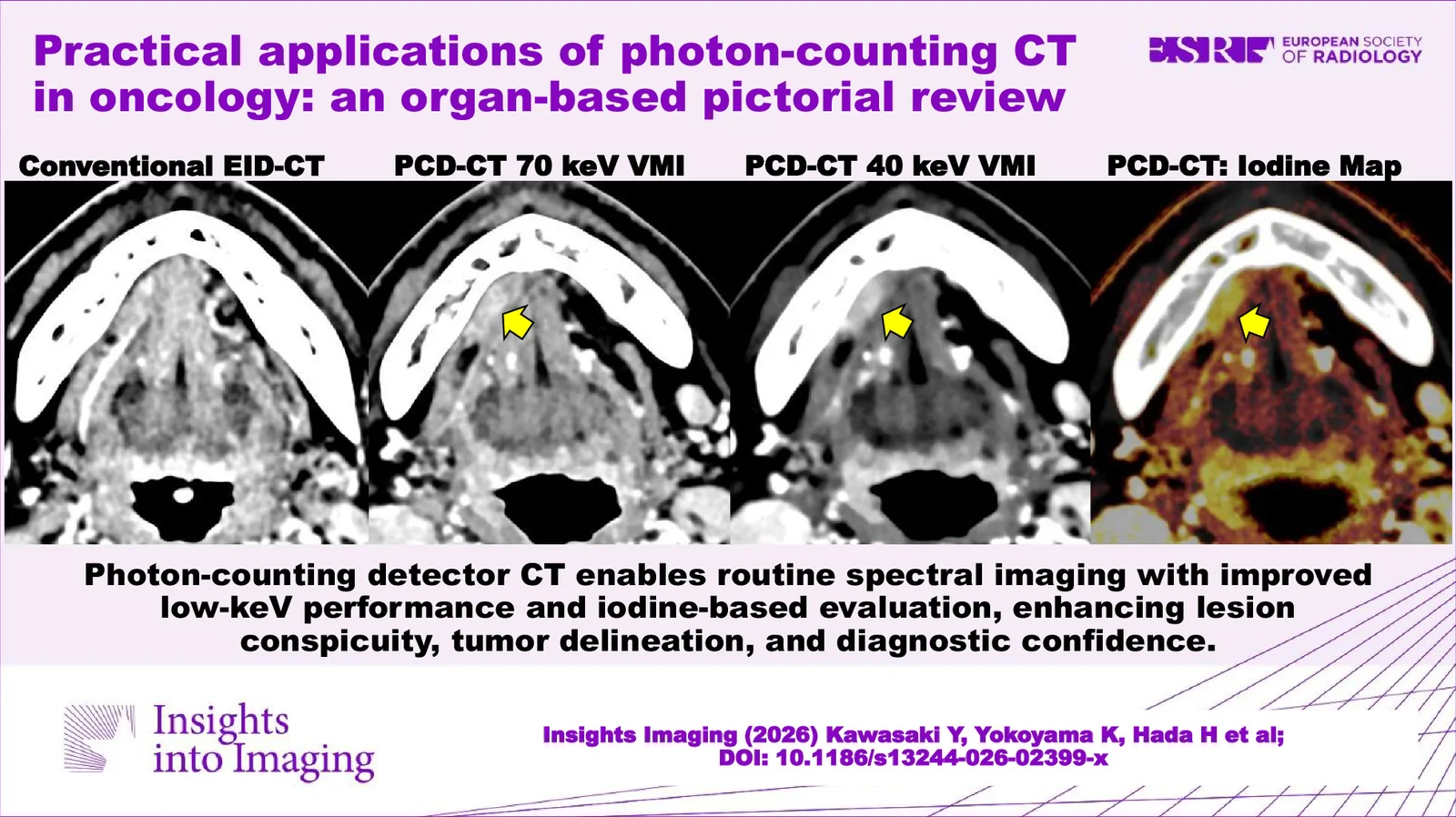

## Introduction

Computed tomography (CT) plays an essential role in oncologic imaging for tumor detection and staging, treatment planning, and response assessment. Due to its wide availability, high spatial resolution, and rapid acquisition, contrast-enhanced CT (CECT) remains the first-line imaging modality for several malignancies.

The clinical value of spectral imaging in oncologic CT has been well established via extensive research using dual-energy CT (DECT) [1–7]. Previous studies have shown that spectral techniques—including low-keV virtual monoenergetic imaging (VMI), iodine mapping, and material decomposition—can improve tumor conspicuity, facilitate differentiation between tumors and surrounding tissues, and provide functional information related to tumor vascularity and treatment response [1, 2]. These advancements indicate that spectral imaging may help overcome the limitations of conventional single-energy CT in oncology.

Despite strong evidence supporting DECT, broader adoption of energy-integrating detector computed tomography (EID-CT)-based spectral imaging has been constrained by low-keV image noise [8] and workflow barriers, including dedicated acquisition mode selection, protocol complexity, and vendor-dependent variability [9–12].

By detecting and counting individual X-ray photons and assigning them to discrete energy bins, photon-counting detector CT (PCD-CT) enables true spectral imaging with improved energy resolution and significantly reduced electronic noise [13–20]. In particular, these physical advantages may be beneficial for low-keV imaging, where the contrast-to-noise ratio is critical for tumor visualization and local staging [21–26].

The recent clinical introduction of whole-body PCD-CT systems has renewed interest in spectral CT for oncologic imaging. Compared with conventional EID-CT-based DECT, PCD-CT routinely acquires multi-energy datasets without increasing protocol complexity and enables the retrospective reconstruction of spectral images from standard CECT [27, 28]. This access to diagnostically relevant spectral information, even when spectral imaging was not explicitly requested at scanning, may expand the clinical applicability of spectral CT [29]. Consequently, radiologists may apply spectral analysis when unexpected diagnostic questions arise, thereby providing additional clinically relevant information to referring physicians.

This pictorial review synthesizes the published DECT literature and emerging PCD-CT evidence on spectral CT in oncology, with emphasis on practical image interpretation, workflow considerations, and representative imaging examples. Despite the established value of DECT-based spectral imaging, important practical questions remain regarding how the advantages of PCD-CT can be translated into routine oncologic imaging.

The current review aims to illustrate how PCD-CT may expand the clinical use of spectral imaging in oncology beyond conventional DECT. The institutional cases were drawn from a study approved by the Institutional Review Board of the Institute of Science Tokyo (Approval No. I2024-084, approved on November 14, 2024).

Via an organ-based pictorial review, we present the practical applications of low-keV VMI, iodine-based reconstructions, and quantitative parameters that may enhance tumor detection, delineation, and diagnostic confidence in routine practice.

According to the framework proposed by Martí-Bonmatí et al, this review synthesizes a heterogeneous, evolving, and early-stage evidence base with variable maturity across applications, as summarized in Table 1.

**Table 1:** Summary of review methodology and evidence level

Item	Description for the present review
Article type	Critical/educational pictorial review
Review design	Narrative, organ-based review integrating published literature and representative institutional cases
Quantitative synthesis	Not performed
Meta-analysis	Not performed
Formal systematic review methodology	Not performed
Original patient-level data	Not included
Main evidence source	Published literature and representative institutional clinical cases
Evidence level	Narrative-review level; heterogeneous and evolving evidence base without a formal systematic review or quantitative synthesis

## Technical foundations of PCD-CT and spectral imaging

### From EID-CT to PCD-CT

**Fig. 1:**
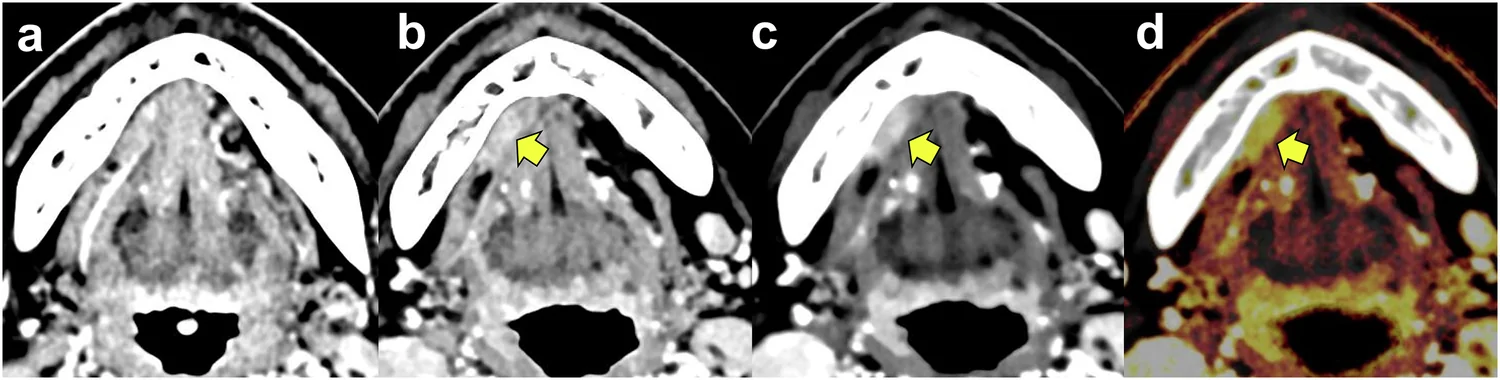
Enhanced conspicuity of floor-of-mouth carcinoma on photon-counting detector CT (PCD-CT) versus energy-integrating detector CT (EID-CT). Floor-of-mouth squamous cell carcinoma evaluated by contrast-enhanced CT. **a** Contrast-enhanced EID-CT shows effacement of the normal fat plane, but lesion margins are poorly delineated due to similar attenuation to adjacent soft tissue. **b** PCD-CT reconstructed as a 70-keV virtual monoenergetic image (VMI) shows more conspicuous enhancement. **c** A 40-keV VMI further increases iodine-related contrast, improving boundary definition. **d** Iodine overlay image further enhances lesion conspicuity. Yellow arrows indicate the tumor

In conventional EID-CT, X-ray photons are converted into visible light and integrated over time, underweighting low-energy photons [27], which makes low-keV images vulnerable to noise [14]. In contrast, PCD-CT directly detects individual photons and assigns them to energy bins, thereby preserving low-energy information while reducing electronic noise [27, 28]. These features support practical low-keV VMI, iodine-based quantitative imaging, and material decomposition, thereby improving lesion conspicuity and soft-tissue contrast in oncologic imaging (Fig. 1) [18, 27, 30].

### Differences in PCD-CT systems

PCD-CT systems vary in detector material and system configuration, resulting in differences in spectral performance, spatial resolution, detector coverage, and temporal resolution [19, 31–36]. Current or prototype whole-body PCD-CT systems include cadmium telluride (CdTe)-, Si-, and cadmium zinc telluride (CZT)-based detector approaches [17, 28, 32]. Most currently available oncologic evidence is derived from CdTe-based clinical systems, reflecting earlier clinical availability rather than definitive superiority over Si- or CZT-based approaches. Therefore, findings from CdTe-based systems should not be assumed to generalize fully across detector platforms without further validation. In oncologic imaging, temporal resolution is generally less important than image noise characteristics, contrast-to-noise ratio, spatial resolution, and spectral performance [29, 31, 37, 38].

## Spectral image interpretation and practical workflow in oncologic imaging

Spectral CT enables the reconstruction of multiple image types from energy-resolved data, thereby providing diagnostic information beyond conventional morphologic assessment.

### Virtual monoenergetic imaging and iodine-related contrast enhancement

VMI plays an essential role in oncologic spectral imaging. Low-keV images, particularly around 40 keV, enhance iodine-related contrast between lesions and surrounding tissues [39].

Although low-keV imaging in EID-CT-based DECT has been limited by image noise [5], PCD-CT enables routine 40-keV VMI reconstruction by reducing electronic noise and improving energy separation [21, 23, 24]. Accordingly, based on DECT-derived and preliminary PCD-CT evidence, 40-keV VMI may serve as a practical initial spectral reconstruction for tumor detection, margin delineation, and assessment of local invasion.

High-keV VMIs, typically above 110 keV, reduce beam hardening and metal artifacts and are used selectively in specific clinical scenarios, such as head and neck imaging with dental hardware, especially when combined with iterative metal artifact reduction techniques [40–44].

### Iodine-based images for tissue characterization and quantification

Iodine-based images, including iodine maps and overlay images, provide complementary information by visualizing the contrast distribution [45–49]. Their primary value lies where morphologic assessment alone is insufficient, such as in differentiating enhancing tumor tissue from non-enhancing structures, including atelectasis, necrosis, or cystic components [46, 47, 50, 51].

In cystic lesions, iodine maps facilitate identification of enhancing solid components, contributing to malignancy risk stratification, as exemplified by pancreatic intraductal papillary mucinous neoplasms and Bosniak-classified renal cysts [52–54]. Iodine-based images also enable quantitative analysis of iodine concentration, which has been reported as a potential imaging biomarker for tumor characterization and treatment response assessment [55–60].

### Other spectral reconstructions and workflow considerations

Virtual non-contrast (VNC) images may approximate true non-contrast images and contribute to protocol simplification and radiation dose reduction in selected settings. In dynamic CECT studies, VNC images may allow omission of an additional true non-contrast acquisition without compromising diagnostic information in appropriately selected patients [61–64].

However, VNC images should be interpreted with caution in specific scenarios. Because iodine-related signals are digitally removed, substances with similar attenuation characteristics, such as lipiodol accumulation after transarterial chemoembolization for hepatocellular carcinoma (HCC), may also be suppressed. Accordingly, prior treatment history should be reviewed before relying on VNC images, particularly in post-treatment liver imaging.

Other spectral parameters, including effective atomic number (Z_eff) [65], fat fraction [66–68], and electron density (ED) maps [69], represent emerging tools for tissue characterization and radiation therapy planning [70], although current evidence supporting their routine use in oncologic imaging remains limited. Extracellular volume (ECV) estimation has also been explored; while its role in tumor assessment is not yet established, it may be useful for evaluating background organ conditions, such as liver fibrosis, and treatment-related organ injury [71–73].

For spectral CT to be effective in routine oncology practice, spectral images must be readily accessible within a seamless workstation environment. Low-keV VMIs (approximately 40 keV) may be reviewed routinely alongside conventional images, with iodine-based images generated selectively if specific diagnostic questions arise. This pragmatic workflow enables spectral CT to enhance diagnostic performance without increasing interpretation complexity and forms the basis for the organ-based applications discussed in the following sections.

## Organ-based clinical applications of spectral CT in oncologic imaging

The clinical value of spectral CT in oncology becomes most evident when applied to specific organs and tumor types. Across malignancies, the primary contributions of spectral imaging include improved lesion conspicuity [24], more accurate delineation of tumor extent [23], and enhanced tissue characterization beyond conventional morphologic assessment [74].

In PCD-CT spectral imaging, low-keV VMIs may serve as the principal image set for tumor detection and local staging in selected contexts [21, 23, 26], while iodine-based images are used selectively to address specific diagnostic questions, such as differentiating an enhancing tumor from non-enhancing tissue [46]. The following sections review the current evidence and emerging applications of spectral CT in oncologic imaging using an organ-based framework. A cross-organ, evidence-aware summary of these applications is provided in Table 2. Evidence-level wording in Table 2 and the organ-specific subsections is aligned with the framework proposed by Martí-Bonmatí et al, while distinguishing DECT-derived evidence, preliminary PCD-CT data, institutional experience, and investigational applications.

**Table 2:** Evidence-aware summary of spectral CT applications in oncology

Organ/system	Representative applications	Main evidence source	PCD-CT evidence maturity	Evidence-aware clinical interpretation
Head and neck oncology	Tumor conspicuity, local extension, extranodal extension assessment, and dental artifact reduction	DECT-derived evidence with emerging PCD-CT studies	Partially established	Supports local-staging confidence and treatment-planning support; quantitative applications remain less standardized.
Thoracic oncology	Tumor–atelectasis separation, pleural invasion assessment, iodine-density analysis, ED-based assessment	Primarily DECT-derived, with increasing PCD-CT data	Partially established	Quantitative tools are promising but are currently supported mainly by early PCD-CT clinical experience.
Breast oncology	Enhancing tumor visualization, lesion extent assessment, and nodal evaluation during systemic CT staging	Preliminary PCD-CT and DECT-derived studies	Emerging	Potential adjunct when MRI is contraindicated, unavailable, or limited.
Hepatobiliary and pancreatic oncology	HCC enhancement assessment, PDAC conspicuity, tumor–vessel relationship, recurrence assessment	Established DECT evidence for low-keV imaging; emerging iodine quantification and PCD-CT data	Well established	Promising for PDAC and liver-lesion assessment; reproducibility and outcome-based value require further study.
Genitourinary and gynecologic oncology	Renal/cystic lesion characterization, adrenal assessment, bladder/prostate/gynecologic enhancement evaluation	Mostly DECT-derived, with preliminary PCD-CT observations	Emerging	Supports active exploration in selected genitourinary and gynecologic oncology workflows.
Gastrointestinal oncology	Rectal cancer T staging, colorectal lesion conspicuity, peritoneal metastasis detection, gastric/esophageal tumor assessment	Preliminary PCD-CT data for rectal cancer; broader GI evidence mainly DECT-derived	Partially established	PCD-CT may expand CT’s role in local assessment across GI malignancies.
Hematologic malignancies and bone marrow imaging	VNCa-based marrow assessment, myeloma infiltration, and subtle osseous lesion detection	Mainly DECT VNCa studies, particularly in myeloma	Investigational	Less mature than low-keV or iodine-based imaging, but promising for quantitative PCD-CT material assessment.
CNS oncology	Lesion-to-brain contrast, enhancement quantification, recurrence versus radiation injury, radiotherapy planning	Predominantly DECT-derived, with limited direct PCD-CT data	Investigational	One of the least mature areas, but a worthwhile frontier for high-resolution, low-noise quantitative CT.
Occult cancer/CUP	Initial whole-body evaluation, subtle primary lesion detection, lesion characterization, triage for advanced imaging	Promising DECT-derived evidence	Perspective/hypothesis-generating	Retrospective spectral reconstruction from routine PCD-CT CECT may support broader CUP problem-solving.

*CECT* contrast-enhanced computed tomography, *CNS* central nervous system, *CUP* cancer of unknown primary, *DECT* dual-energy computed tomography, *ED* electron density, *GI* gastrointestinal, *HCC* hepatocellular carcinoma, *MRI* magnetic resonance imaging, *PCD-CT* photon-counting detector computed tomography, *PDAC* pancreatic ductal adenocarcinoma, *VNCa* virtual non-calcium, *VMI* virtual monoenergetic imaging

### Head and neck oncology

Head and neck cancers present diagnostic challenges due to complex anatomy, close proximity to critical soft tissues and neurovascular structures, and the frequent presence of dental hardware [75–79]. Accurate tumor detection and precise delineation of local tumor extent are therefore essential for staging, treatment planning, and selection of surgical or organ-preserving strategies [80–85].

Spectral CT has demonstrated clinical utility across various head and neck malignancies, including cancers of the tongue, oral cavity, salivary glands, pharynx, larynx, and thyroid [26, 86, 87]. 40–45-keV images improve visualization of enhancing tumors and the assessment of tumor extent and invasion (Fig. 1) [26, 82]. These properties support more confident staging, including imaging-detected extranodal extension, and surgical planning [26, 88]. High-keV VMI can be selectively applied to reduce beam-hardening artifacts and assist in the evaluation of osseous invasion [40–44].

**Fig. 2:**
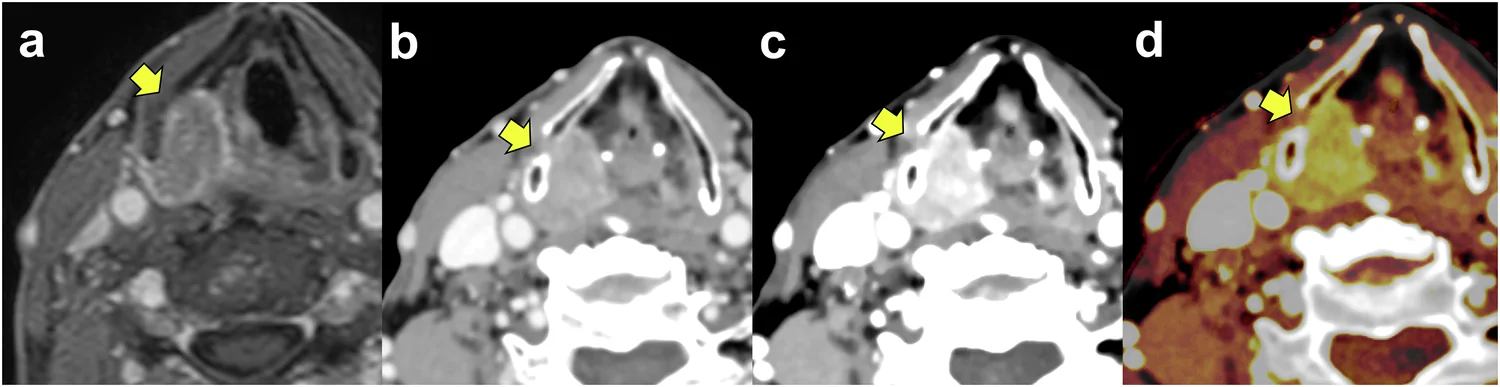
Differentiation of tumor abutment at the thyroid foramen from thyroid cartilage invasion in laryngeal cancer using photon-counting detector CT (PCD-CT). Laryngeal cancer evaluated by MRI and PCD-CT. **a** Axial contrast-enhanced T1-weighted MRI shows an enhancing tumor extending laterally toward the thyroid cartilage (arrow). **b** Axial 70-keV virtual monoenergetic image (VMI) shows subtle tumor extension adjacent to the thyroid cartilage (arrow). **c** Corresponding 40-keV VMI more clearly depicts the enhancing tumor abutting the thyroid foramen without extension through the foramen or cartilage destruction (arrow). **d** Iodine overlay image even more clearly delineates tumor enhancement overlying the inner opening of the thyroid foramen without extension beyond it, increasing confidence that thyroid cartilage invasion is absent (arrow)

Iodine-based images support assessment of tumor enhancement and increase diagnostic confidence in defining local tumor extent [89]. In glottic cancer, a clearer depiction of normal anatomic structures, such as the thyroid foramen, facilitates differentiation between tumor abutment at the foramen and true invasion of the thyroid cartilage (Fig. 2) [90–92].

In DECT, quantitative spectral parameters, including iodine concentration and Z_eff, have been investigated for assessing nodal metastasis, characterizing tumors, and detecting recurrence; however, reported thresholds and analysis strategies remain heterogeneous, and further standardization is required [87, 93, 94].

Overall, spectral CT—centered on low-keV and iodine-based imaging—may improve tumor conspicuity and local staging in head and neck oncology and can provide complementary CT-based information when MRI is unavailable, contraindicated, or limited [95–97]. In PCD-CT, low-energy imaging offers favorable noise properties, extending the clinical applicability of low-keV techniques beyond conventional DECT. Quantitative spectral parameters such as iodine concentration and Z_eff, however, require further validation [26, 82].

Current evidence for head and neck applications remains emerging, combining preliminary PCD-CT evidence with mainly DECT-derived quantitative support; validation and quantitative thresholds require further standardization.

### Thoracic oncology

Lung cancer imaging requires accurate assessment of primary tumor extent, pleural involvement, and differentiation from non-neoplastic lung changes [74]. Central lung cancers complicated by post-obstructive atelectasis or pneumonia, subtle pleural invasion, and indeterminate mediastinal lymph nodes remain major sources of diagnostic uncertainty on conventional EID-CT [17, 50, 98].

Low-keV reconstructions improve the assessment of lung tumors [50, 74]. In PCD-CT, these images exhibit stable noise characteristics, allowing clearer depiction of tumor margins and local extent, including pleural invasion, particularly when attenuation differences between the tumor and adjacent lung parenchyma are limited [99].

**Fig. 3:**
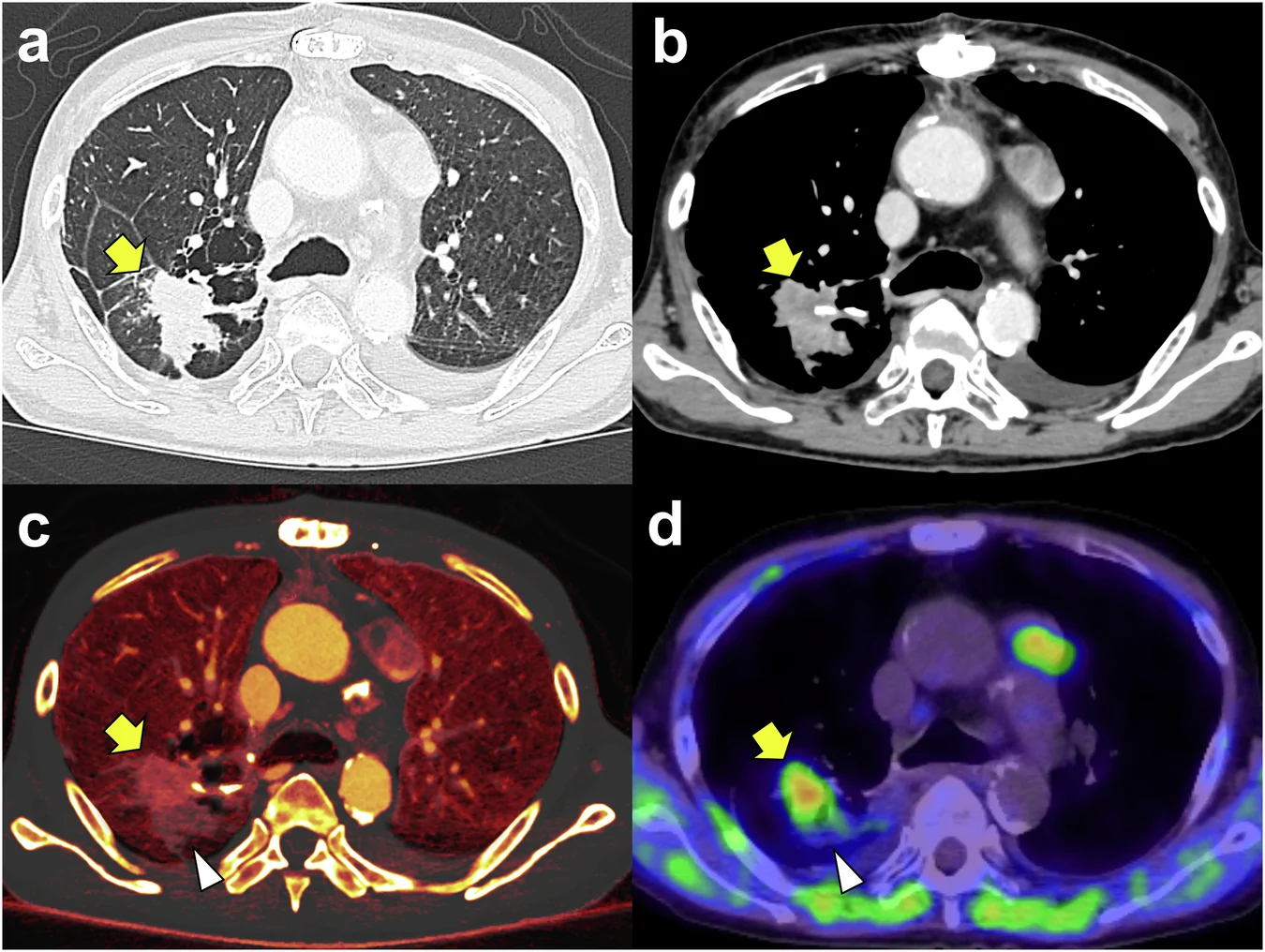
Assessment of intratumoral viability in lung cancer using iodine mapping. Lung cancer evaluated by CT with iodine mapping. **a** Lung-window CT shows a right upper lobe mass (arrow). **b** Contrast-enhanced CT shows apparently homogeneous enhancement (arrow). **c** The iodine map demonstrates an iodine-negative dorsal region (arrow), suggesting poor enhancement; adjacent structures show preserved iodine uptake (arrowhead). **d** ^18^F-fluorodeoxyglucose (FDG) PET/CT shows no FDG uptake in the corresponding dorsal region (arrow), supporting a non-viable component

Iodine-based images selectively highlight viable tumor enhancement (Fig. 3) [100–102]. Iodine maps and overlay images enable more confident distinction between enhancing tumors and adjacent atelectasis or post-obstructive consolidation [50, 51], improving tumor size measurement, staging accuracy, and radiotherapy target delineation [74, 101]. They may also assist in distinguishing postoperative fibrosis from local recurrence and in suggesting tumor aggressiveness or type, as well as primary versus metastatic disease [101, 103, 104].

ED imaging derived from DECT complements the assessment of ground-glass nodules and invasive components [74, 105]. By enabling low-keV and iodine-based imaging with clinically acceptable image quality, PCD-CT provides a practical spectral platform for lung cancer evaluation.

Current evidence for thoracic oncology applications is primarily DECT-derived, but PCD-CT-specific data are increasing. Iodine density imaging and ED-based assessment may offer promising quantitative tools for tumor characterization, treatment response evaluation and radiotherapy planning as the evidence base continues to evolve.

### Breast oncology

Traditional breast imaging relies on mammography, ultrasonography, and contrast-enhanced MRI (CE-MRI) for detection and local staging, whereas CT has mainly been used for systemic evaluation. Recent advances in spectral imaging and PCD-CT have expanded the potential role of CECT in breast oncology [106–110].

Low-keV VMI improves visualization of enhancing breast tumors within fibroglandular tissue, particularly in dense breasts and for assessing ductal and invasive components [108, 111–113]. Compared with conventional CT, preliminary PCD-CT and DECT studies suggest that low-keV reconstructions may improve lesion-size estimation and reduce underestimation of intraductal spread, with MRI and pathology serving as reference comparators [110, 111].

Iodine-based images provide complementary value by highlighting viable tumor tissues and metastatic lymph nodes. Spectral parameters such as iodine density and spectral slope improve noninvasive assessment of sentinel and regional nodal involvement, with potential to reduce unnecessary sentinel lymph node biopsy in selected patients [107, 108, 114, 115].

PCD-CT provides high spatial resolution and spectral performance, enabling improved evaluation of multifocal disease, subtle invasive components, and treatment-related changes [106–108]. As CT is acquired in the supine position, PCD-CT offers anatomic information that closely matches the surgical field, facilitating preoperative mapping and breast-conserving surgical planning [106, 111].

**Fig. 4:**
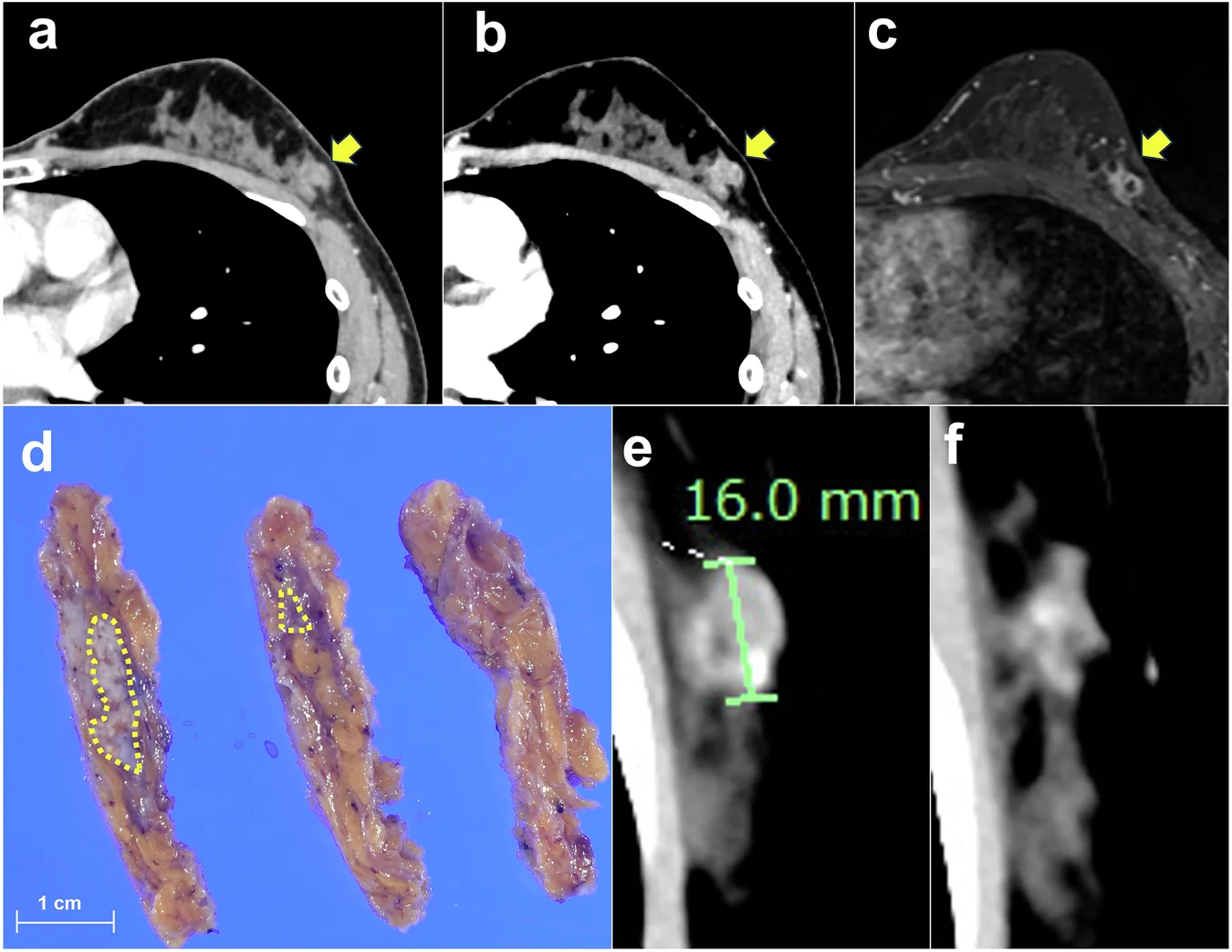
Depiction of ductal carcinoma in situ (DCIS) using photon-counting detector CT (PCD-CT). **a** Axial 70-keV virtual monoenergetic image (VMI) demonstrates subtle contrast enhancement with limited lesion conspicuity (arrow). **b** Corresponding axial 40-keV VMI shows increased conspicuity and clearer delineation of the lesion due to enhanced iodine-related attenuation (arrow). **c** Early-phase contrast-enhanced T1-weighted MRI demonstrates enhancement patterns comparable to those observed on low-keV PCD-CT (arrow). **d** Gross pathological specimens of the resected breast tissue after formalin fixation demonstrate the distribution of DCIS (outlined by yellow dotted lines), corresponding to the enhancing regions on imaging; the maximum pathological extent was measured as 15 mm (scale bar = 10 mm). **e**, **f** Oblique sagittal reconstructions of the 40-keV PCD-CT images, oriented to match the pathological slicing plane, demonstrate a lesion extent of 16.0 mm, which is only slightly larger than that observed in the formalin-fixed pathological specimens, suggesting close agreement with the actual in vivo lesion extent

Preliminary PCD-CT and DECT-derived studies suggest that low-keV reconstructions may improve visualization of enhancing breast tumors during systemic CT staging (Fig. 4) [108, 110, 111]. MRI remains the reference standard for local breast assessment; however, PCD-CT may serve as a complementary adjunct in selected patients, particularly when MRI is contraindicated, unavailable, or limited. Although evidence for breast applications remains emerging, further validation may define how PCD-CT-based spectral CECT can streamline systemic staging, local enhancement assessment, and follow-up imaging in selected clinical scenarios.

### Hepatobiliary and pancreatic oncology

Hepatobiliary and pancreatic malignancies are well suited for spectral CT because diagnosis and staging often depend on subtle enhancement differences and accurate vascular assessment [116–118]. 40-keV VMIs can increase tumor-to-background contrast and support tumor detection and assessment of extent. In HCC, low-keV imaging enhances the visibility of arterial-phase hyperenhancement and tumor margins, thereby supporting the detection of small lesions and increasing confidence in typical enhancement patterns [71, 119–121]. In pancreatic ductal adenocarcinoma (PDAC), which is typically hypovascular, low-keV imaging improves contrast between enhancing pancreatic parenchyma and tumors, thereby facilitating lesion detection [22, 24, 122], local staging, and the evaluation of tumor–vessel relationships relevant to resectability [123].

Some studies, which are based on visual assessments of lesion conspicuity and image noise, have revealed an optimal energy level of approximately 55 keV [22], slightly higher than 40 keV. This likely reflects the fact that PDAC is conventionally evaluated using multiphasic dynamic CECT as the diagnostic gold standard and that experienced radiologists may already achieve high detection rates with image appearances closer to conventional CT, similar to 70-keV reconstructions [24]. Consequently, although low-keV imaging clearly enhances contrast, its incremental benefit for PDAC detection may be less evident in the hands of experts.

**Fig. 5:**
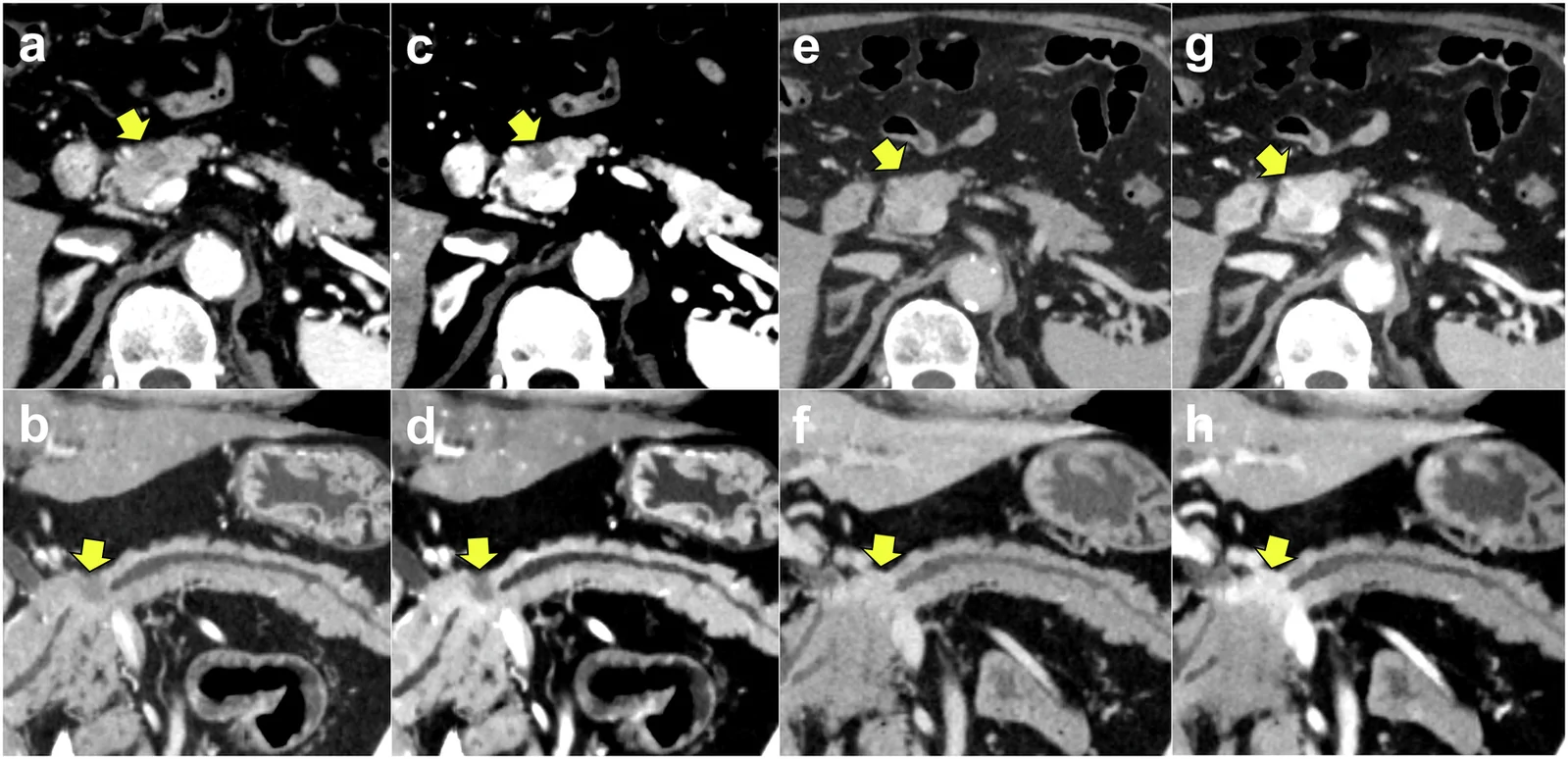
Improved visualization of pancreatic cancer on low-keV photon-counting detector CT. Pancreatic ductal adenocarcinoma evaluated by contrast-enhanced CT. **a**, **b** Arterial-phase 70-keV images show a hypovascular pancreatic head mass (arrows) with abrupt pancreatic duct interruption. **c**, **d** Arterial-phase 40-keV images improve contrast between tumor and enhancing parenchyma (arrows). **e**, **f** Portal venous–phase 70-keV images show subtle delayed tumor enhancement (arrows). **g**, **h** Portal venous–phase 40-keV images depict more conspicuous enhancement (arrows). This example illustrates that low-keV imaging can improve tumor conspicuity across phases, particularly for subtle delayed enhancement

From a practical perspective, PCD-CT may improve the visualization of dynamic enhancement behavior beyond the arterial phase. Washout of HCC from the portal venous to the delayed phases, and delayed enhancement of PDAC can be depicted more clearly with PCD-CT (Fig. 5) [24]. This indicates that lesion characterization may be feasible even with only portal venous or delayed-phase images, implying that HCC or PDAC may be evaluable on CECT examinations acquired for other indications without a dedicated multiphasic protocol.

Iodine maps are most useful when morphologic assessment is equivocal [124]. Iodine maps help differentiate viable tumors from nonenhancing components, such as necrosis and postoperative change. Moreover, they can improve confidence in the assessment of recurrence [125]. Quantitative spectral metrics, including iodine concentration and spectral attenuation parameters, have been investigated as imaging biomarkers of tumor biology, such as aggressive features in HCC [126–128] and the grading or characterization of pancreatic tumors [129–131], including neuroendocrine neoplasms [132–134] and cystic lesions in which the identification of enhancing solid components is clinically important [1].

Overall, spectral CT—centered on low-keV VMI with the selective use of iodine-based analysis—may provide a practical CT-based adjunct for detection, staging, and follow-up of hepatobiliary and pancreatic cancers. PCD-CT may further improve image quality and workflow feasibility. Current evidence is predominantly DECT-derived; however, emerging PCD-CT data for PDAC and HCC cohorts are promising. Further studies should focus on quantitative reproducibility and outcome-based clinical value.

### Genitourinary oncology (kidney, urinary tract, gynecologic malignancies)

**Fig. 6:**
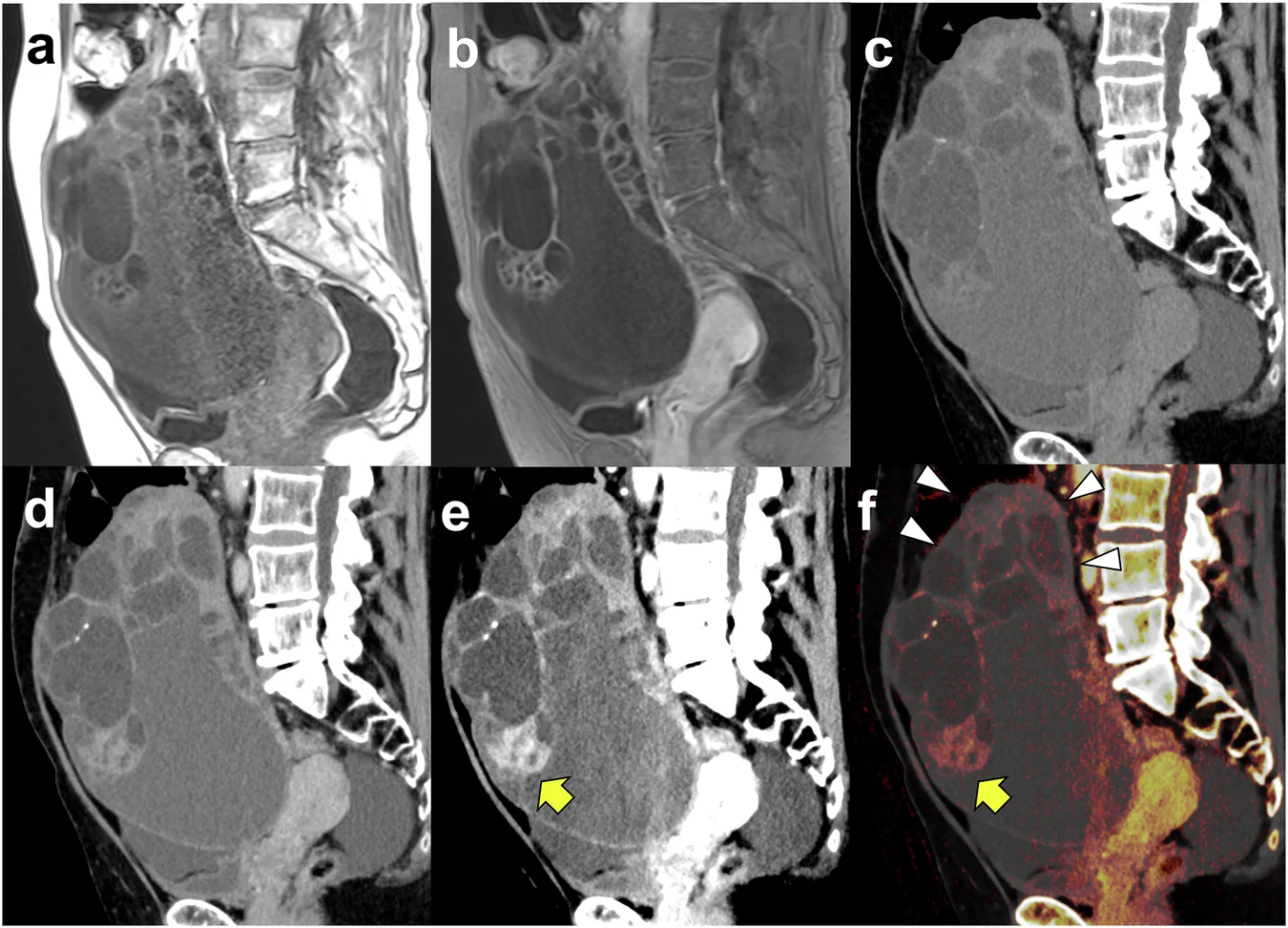
Ovarian mucinous borderline tumor assessed with spectral CT. Ovarian mucinous borderline tumor evaluated by MRI and photon-counting detector CT. **a** Sagittal T1-weighted MRI shows a multilocular mass with variable T1 signal. **b** Fat-suppressed contrast-enhanced T1-weighted MRI suggests septal enhancement, but assessment is limited by intrinsic T1 hyperintensity. **c** Unenhanced CT shows relatively high attenuation of mucinous components. **d** Contrast-enhanced 70-keV virtual monoenergetic image (VMI) shows mild enhancement of septa/solid components. **e** 40-keV VMI increases the enhancement conspicuity of a ventral solid component (arrow). **f** Iodine map confirms iodine uptake in the ventral component (arrow) and helps distinguish enhancing from non-enhancing components (arrowheads)

In renal cell carcinoma, spectral CT improves the characterization of enhancing renal masses and differentiation from cystic or minimally enhancing lesions [1, 135]. In particular, iodine-based images are valuable for validating true enhancement and the risk stratification of cystic renal lesions, including Bosniak classification. Similarly, in ovarian tumors, iodine concentration and extracellular volume maps facilitate reliable differentiation between cystic components and enhancing solid portions, which is especially advantageous in hemorrhagic lesions, where intrinsic T1 hyperintensity is challenging to distinguish from true enhancement on MRI [136–138]. In both renal and ovarian tumors, iodine maps improve confidence in identifying viable tumor components within complex cystic masses (Fig. 6).

Adrenal tumors are among the most established applications of spectral CT in the genitourinary domain [100, 139–141]. By combining VNC images with iodine-based parameters, spectral CT enables reliable differentiation between adrenal adenoma and metastasis, including lipid-poor adenomas that are challenging to identify on conventional CT [100, 142]. Beyond metastatic disease, although current evidence is limited to small cohorts, previous studies using DECT have shown that low-keV images, iodine maps, and related spectral parameters may provide complementary information to differentiate pheochromocytoma from adrenal adenoma [143]. In this context, PCD-CT, with its improved energy resolution and noise reduction, further enhances quantitative iodine assessment and lesion conspicuity, which allows streamlined, dose-efficient evaluation while reducing the need for additional unenhanced scans or MRI.

**Fig. 7:**
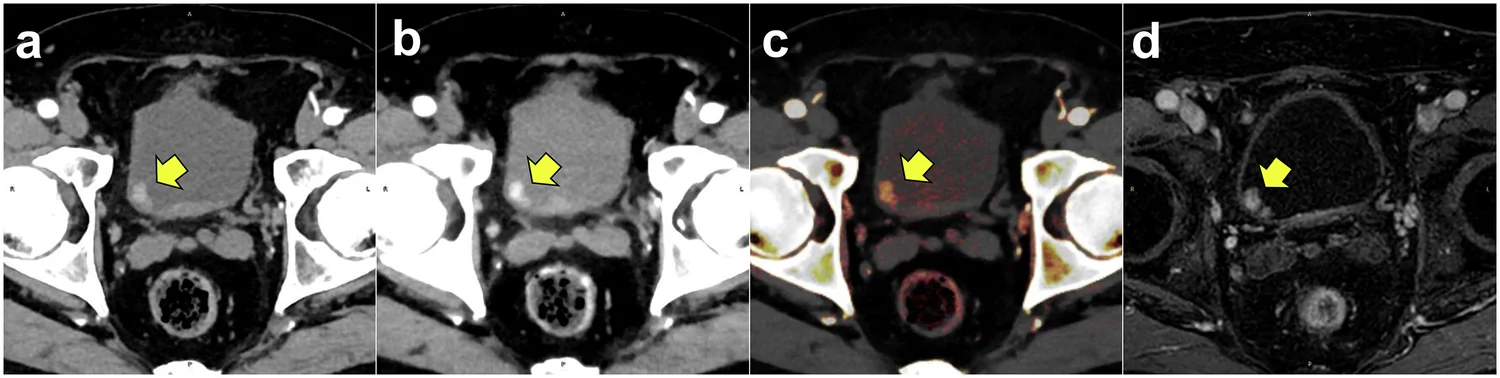
Bladder cancer evaluated by photon-counting detector CT. Bladder cancer evaluated by photon-counting detector CT and MRI. **a** Contrast-enhanced 70-keV virtual monoenergetic image (VMI) shows limited conspicuity of a bladder wall mass (arrow). **b** Corresponding 40-keV VMI improves lesion conspicuity (arrow). **c** The iodine map clearly demonstrates iodine uptake within the tumor (arrow), aiding assessment of possible muscle layer involvement. **d** Fat-suppressed contrast-enhanced T1-weighted MRI shows concordant tumor extent (arrow). Transurethral resection confirmed urothelial carcinoma, pathological stage pTa, low grade (G2), without subepithelial invasion

In bladder [144, 145] and prostate cancer [146, 147], low-keV imaging provides improved enhancement conspicuity. In the illustrative PCD-CT/MRI case shown in Fig. 7, 40-keV VMI and iodine mapping increase tumor conspicuity relative to 70-keV images and show concordant tumor extent with contrast-enhanced T1-weighted imaging (CE-T1WI); this observation should be interpreted as preliminary institutional experience. Accordingly, contrast-enhanced PCD-CT may provide complementary local assessment information in selected patients, but should not be regarded as a replacement for dedicated pelvic MRI.

In gynecologic malignancies, including cervical and endometrial cancers, low-keV imaging may contribute to a more accurate assessment of local tumor extent. A better contrast between tumor and adjacent myometrium or cervical stroma on 40-keV images may support the assessment of invasion depth and parametrial involvement [21, 148–150], indicating a complementary role for spectral CT in T-stage evaluation.

Although high-resolution T2-weighted imaging and diffusion-weighted imaging remain essential for dedicated local staging, PCD-CT should not be viewed as a replacement for MRI. Rather, it may provide supplementary enhancement-based information that partly overlaps with CE-T1WI. In selected clinical scenarios, integrating this local enhancement assessment with systemic staging or follow-up CECT could potentially simplify the imaging workflow and reduce the overall imaging burden. Overall, current evidence for genitourinary applications remains mostly DECT-derived, with PCD-CT-specific local-staging observations still limited to preliminary and institutional experience. Nevertheless, these findings support active exploration of PCD-CT in selected genitourinary and gynecologic oncology workflows.

### Gastrointestinal oncology

In gastrointestinal oncology, PCD-CT-based spectral imaging has been promising for local tumor assessment, most notably in rectal cancer [23, 151]. Recent studies have shown that 40-keV images enhance tumor-to-wall contrast and clarify the rectal wall layers, enabling reliable discrimination between tumors confined to the wall (≤ T2) and those with extramural extension (≥ T3), a distinction that is important for treatment planning.

**Fig. 8:**
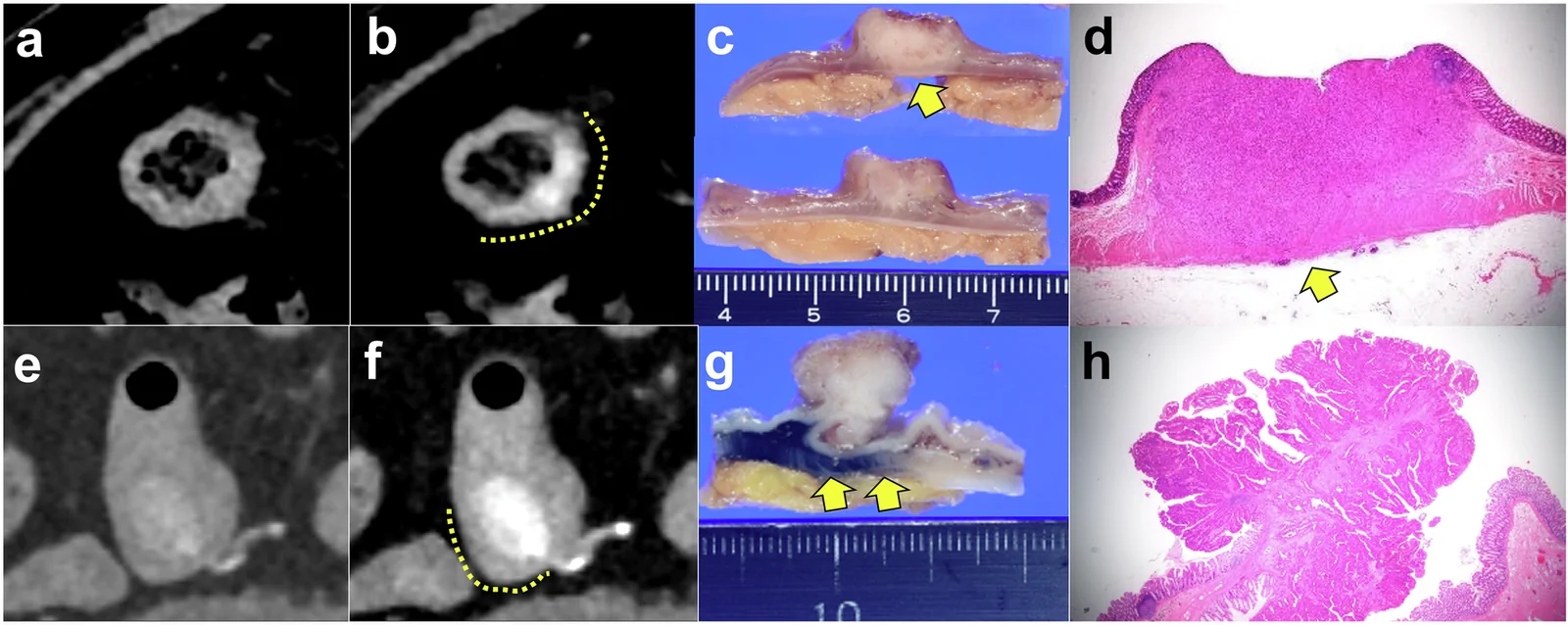
Double colorectal cancers evaluated by low-keV photon-counting detector CT. Synchronous colorectal cancers in the transverse and sigmoid colon. **a**–**d** Transverse colon cancer. **a** 70-keV virtual monoenergetic image (VMI) shows poor lesion conspicuity. **b** 40-keV VMI improves visualization and demonstrates preserved outer bowel wall contour, consistent with cT2 (dotted outline). **c** Gross pathology shows a tumor confined to the bowel wall. **d** Histopathology confirms muscularis propria invasion without serosal involvement (pT2). **e**–**h** Sigmoid colon cancer. **e** 70-keV VMI is inconspicuous. **f** The 40-keV VMI shows the lesion with preserved muscularis propria–equivalent thickness, consistent with cT1b disease (dotted outline). **g** Gross pathology shows a polypoid lesion with preserved muscularis propria (arrows). **h** Histopathology confirms submucosal invasion, leading to a diagnosis of pT1b. Low-keV PCD-CT supports the detection of subtle lesions and the differentiation of cT1 versus cT2 disease

Current PCD-CT-specific evidence in gastrointestinal oncology remains limited to rectal cancer. Extension of these advantages to other colorectal sites remains hypothesis-generating and should be interpreted in light of previous DECT studies [152] and illustrative PCD-CT examples (Fig. 8). Low-keV imaging may enhance mucosal and submucosal contrast, which could facilitate the detection of subtle lesions, including flat or superficially spreading T1 colorectal cancers that may be inconspicuous on conventional CT. DECT studies have also shown that 40-keV images improve the visualization of peritoneal metastases by increasing lesion-to-peritoneum contrast [153, 154].

**Fig. 9:**
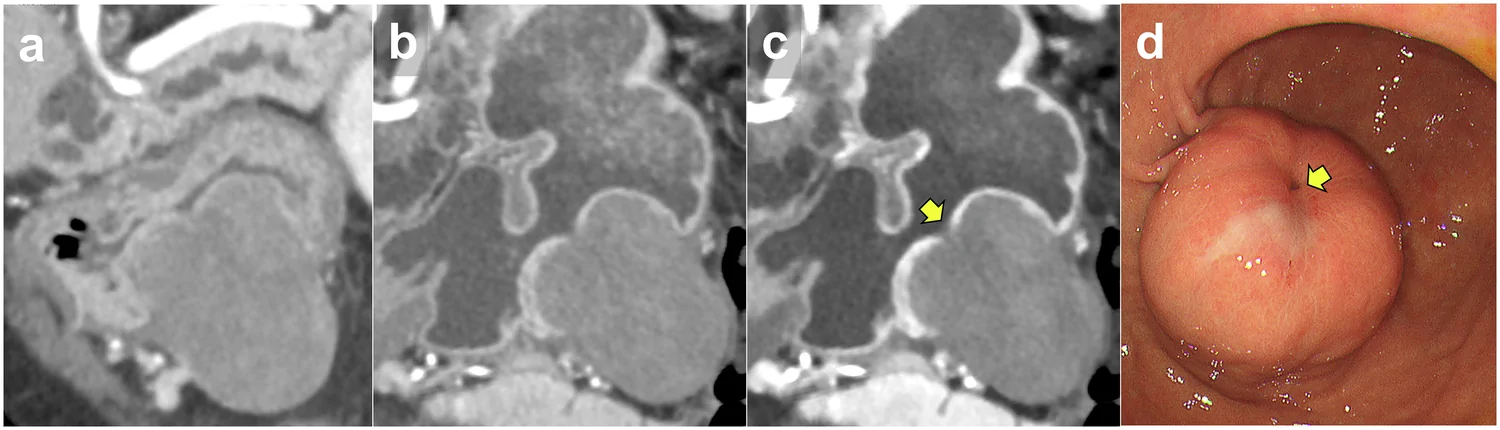
Gastric submucosal tumor detected by photon-counting detector CT. Gastrointestinal stromal tumor (GIST) of the stomach incidentally detected on contrast-enhanced CT. **a** Arterial-phase EID-CT shows limited conspicuity of a submucosal gastric lesion. **b** Photon-counting detector CT (PCD-CT) as 70-keV virtual monoenergetic image (VMI) improves lesion visualization. **c** 40-keV VMI provides the highest conspicuity and depicts focal disruption of the overlying mucosa with a small opening (arrow). **d** Endoscopy confirms a submucosal tumor with a mucosal defect corresponding to CT findings

Beyond the colorectum, low-keV spectral images improve the visual assessment of gastric and esophageal cancers by enhancing lesion-to-wall contrast, thereby supporting evaluation of tumor extent and local invasion [155–157]. Further, prior DECT studies have shown the utility of iodine-based and spectral parameters for assessing nodal status [156], tumor aggressiveness [158–160], prognosis [161], and treatment response in gastric cancer [162–164]. Similarly, these features are beneficial for submucosal tumors, such as gastrointestinal stromal tumors [165], enabling a clearer depiction of lesion localization and luminal relationship (Fig. 9), with additional studies reporting correlations between spectral parameters and Ki-67 expression [160]. Collectively, these observations indicate that spectral CT—particularly with PCD-CT—may extend the role of CT from systemic staging toward a more refined local assessment across gastrointestinal malignancies.

Current evidence for gastrointestinal applications includes preliminary PCD-CT data for rectal cancer, while broader GI applications are mainly supported by DECT-derived evidence. Building on these spectral CT findings, the clinical role of PCD-CT may expand across a wider range of GI malignancies.

### Hematologic malignancies and bone marrow imaging

Hematologic malignancies, such as multiple myeloma and lymphoma, frequently involve the bone marrow, where disease burden may be diffuse or patchy and inconspicuous on conventional CT [166]. Therefore, MRI and ¹⁸F-fluorodeoxyglucose positron emission tomography (PET)/CT remain important for marrow assessment, tumor staging, and response evaluation [166]. However, both modalities have practical limitations in longitudinal care: PET/CT can be difficult to repeat due to cost and cumulative radiation exposure [167]. Meanwhile, although highly sensitive for marrow infiltration, MRI requires longer examination times and may be limited in whole-body assessment [168–170]. Post-treatment marrow signal abnormalities can persist and complicate interpretation [171–173]. In this context, CT-based spectral imaging is gaining attention as a practical adjunct. This is because it can extract marrow-related information from routine CT examinations while simultaneously evaluating extramedullary disease, which may be particularly valuable during follow-up [170, 174, 175].

**Fig. 10:**
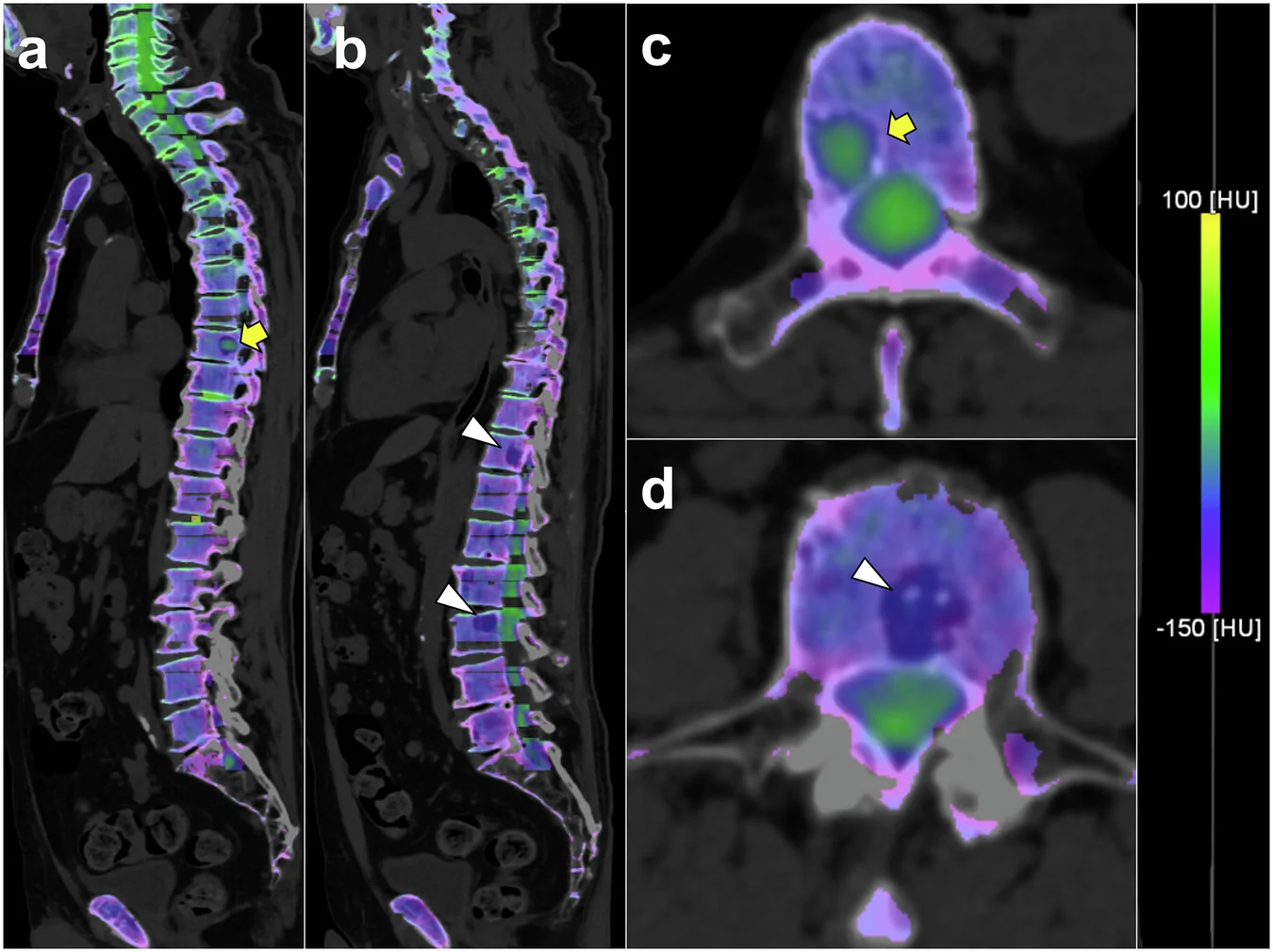
Virtual non-calcium (VNCa) imaging for disease activity in multiple myeloma. Multiple myeloma assessed with VNCa imaging. **a** Sagittal VNCa overlay shows increased attenuation in the T8 vertebral body (arrow), suggesting marrow edema/active disease. **b** Osteolytic lesions are present (arrowheads) without corresponding VNCa increase, suggesting inactive lesions. **c** Axial VNCa at T8 shows focal increased attenuation (arrow). **d** Axial VNCa at T12 shows an osteolytic lesion without VNCa increase (arrowhead). VNCa provides STIR-like marrow contrast over wide anatomical coverage, aiding assessment of lesion activity

Composition-oriented marrow imaging is a key opportunity for spectral CT. DECT studies have shown that virtual non-calcium (VNCa) techniques can suppress trabecular calcium and better depict marrow soft-tissue components, thereby reflecting marrow cellularity and edema-like changes and correlating with MRI-derived marrow findings in cohorts with myeloma [176–179]. By targeting marrow composition rather than relying solely on morphologic lytic change, VNCa-based approaches may enable the earlier recognition of intramedullary disease and a more objective assessment of tumor burden, including cases in which conventional CT underestimates non-lytic or diffuse infiltration (Fig. 10). Spectral CT can also support the evaluation of skeletal complications and treatment-related changes. Current evidence is derived from emergency imaging studies [180, 181] and research on solid tumors such as lung cancer [182]. However, this integrated whole-body CT and marrow imaging approach is readily applicable to hematologic malignancies.

PCD-CT may further strengthen these applications. Clinical evidence on bone marrow imaging with PCD-CT remains limited [183, 184]. Its low-noise characteristics and higher spatial resolution may support high-quality marrow depiction and improved visualization of subtle osseous abnormalities and small lytic lesions, but this application remains preliminary and requires outcome-driven validation. These properties may increase diagnostic confidence and facilitate lower-dose protocols that are better suited for repeated examinations. Overall, CT-based spectral imaging—leveraging VNCa and related spectral reconstructions, with PCD-CT providing additional gains in image quality—offers a practical pathway to incorporate marrow-sensitive information into routine CT workflows, complementing MRI and PET/CT in hematologic oncology. Compared with low-keV imaging and iodine-based reconstructions, evidence for VNCa-based marrow assessment remains less mature and is still largely derived from DECT studies, particularly in myeloma. Nevertheless, marrow imaging may represent a promising field for PCD-CT through quantitative material assessment and high-resolution depiction of subtle osseous and marrow abnormalities.

### Central nervous system oncology

MRI remains the reference standard for central nervous system (CNS) tumor imaging. However, DECT-derived evidence suggests that spectral CT may serve as a complementary modality in selected clinical scenarios by providing quantitative, material-specific information that extends CT beyond purely morphologic assessment [185–188].

DECT-derived VMIs and iodine maps can improve lesion-to-brain contrast and enable the quantitative evaluation of enhancement. In gliomas, iodine concentration and spectral attenuation metrics reflect vascularity and cellularity. Further, they are correlated with tumor grade and proliferative activity, and have potential utility for molecular characterization, such as isocitrate dehydrogenase (IDH) and O6-methylguanine-DNA methyltransferase (MGMT) status, as well as for differential diagnosis, such as distinguishing metastatic lesions from glioblastoma [185–187, 189]. In particular, these features are useful when MRI findings are equivocal or incomplete.

Evidence specific to primary CNS lymphoma remains limited. However, given the characteristic iodine-related patterns reported in systemic and extranodal lymphomas (including orbital lymphoma) [190, 191], iodine-based spectral information may add value to lesion characterization and problem-solving when MRI is equivocal or incomplete. From a detection standpoint, spectral CT is interesting because it can be performed seamlessly alongside routine CECT, thereby supporting integrated assessment without additional dedicated examinations [2, 192].

**Fig. 11:**
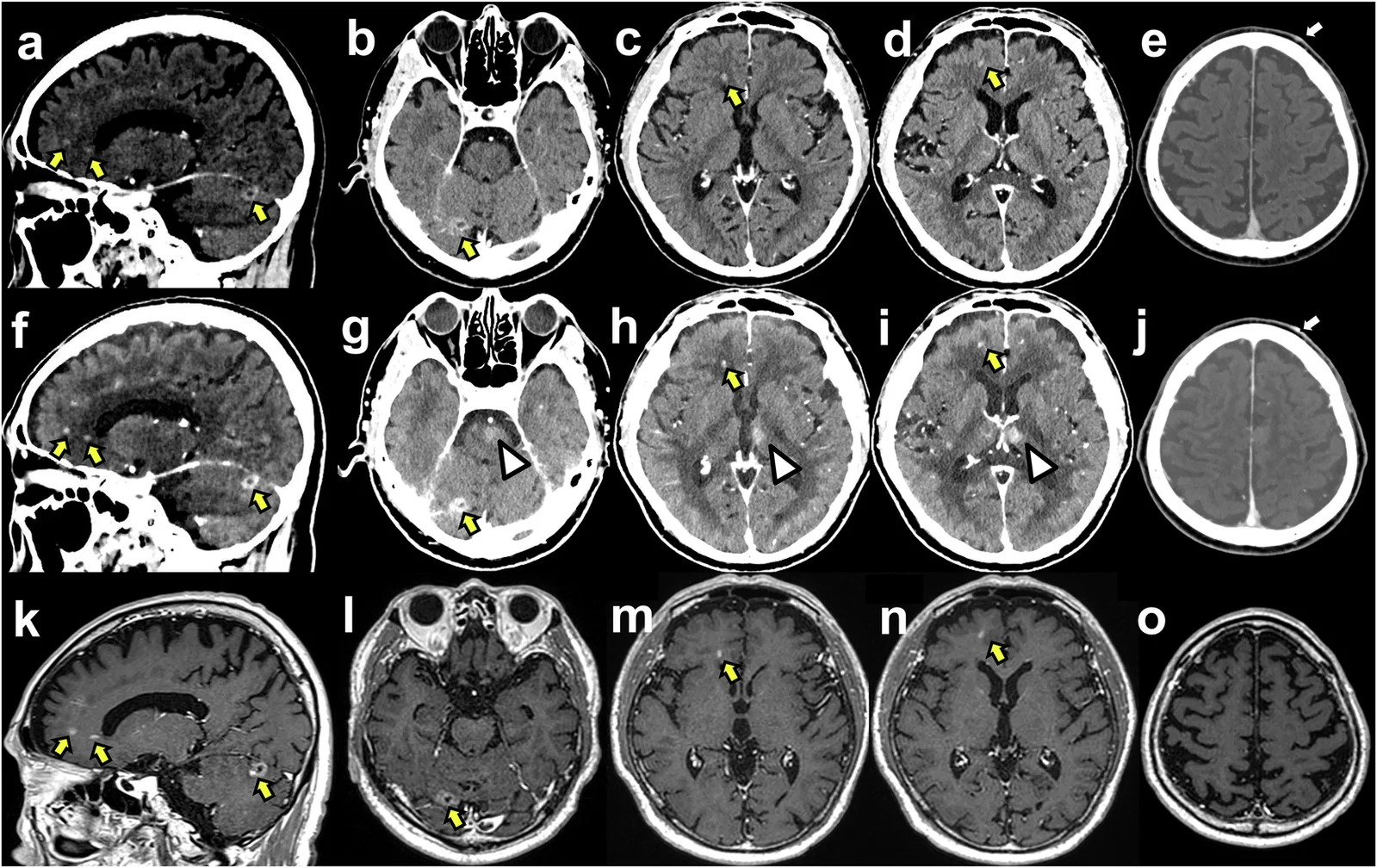
Brain metastases detected by photon-counting detector CT. Brain metastases evaluated by photon-counting detector CT (PCD-CT) and MRI. **a**–**e** 70-keV virtual monoenergetic image (VMI) shows small enhancing lesions with limited conspicuity (arrows). **f**–**j** Corresponding 40-keV VMI markedly improves the conspicuity of the same lesions (arrows). **k**–**o** Contrast-enhanced T1-weighted MRI confirms metastases at corresponding locations (arrows). Triangle arrowheads indicate potential imaging artifacts near the isocenter that were more commonly observed with earlier hardware versions but have been addressed in more recent hardware versions

**Fig. 12:**
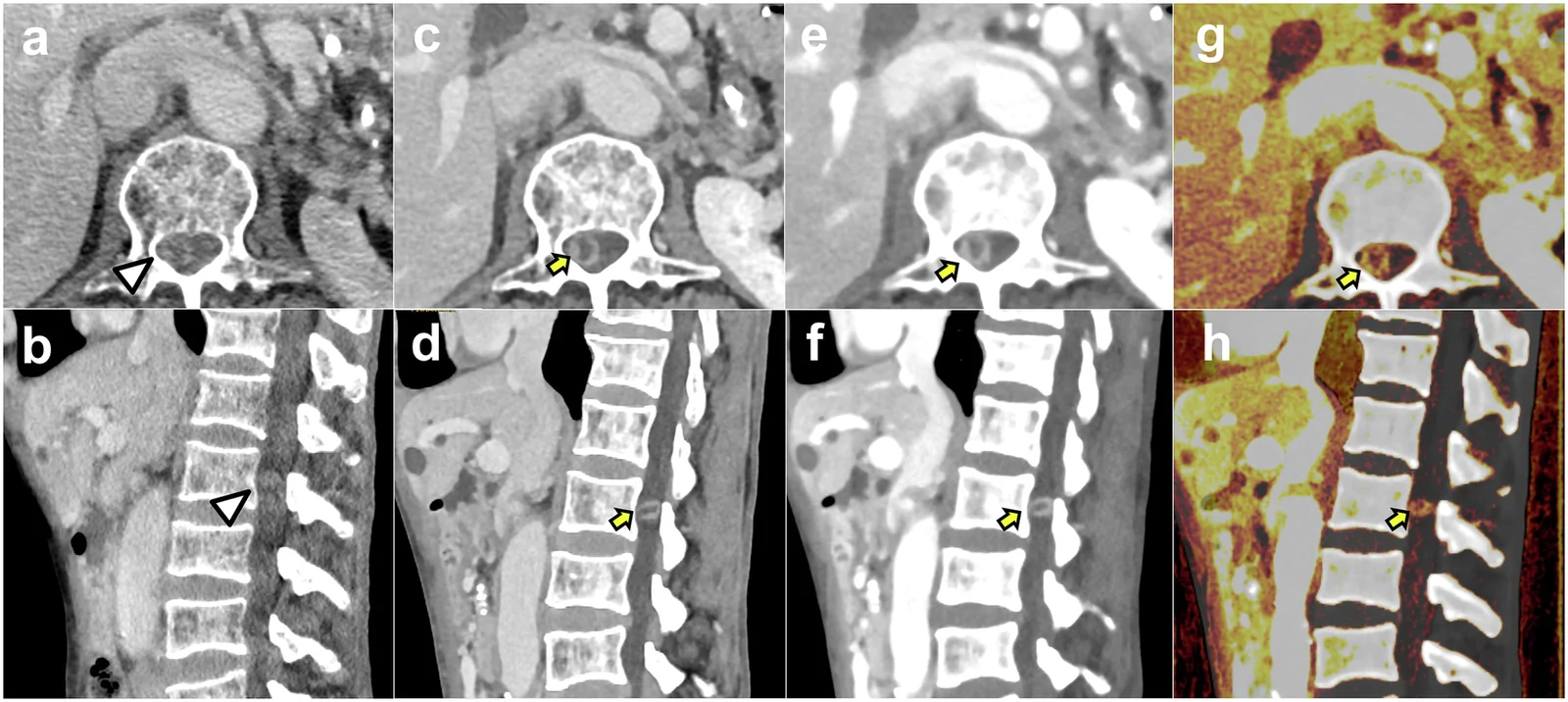
Spinal tumor detected by photon-counting detector CT. A spinal lesion detected on photon-counting detector CT (PCD-CT) and characterized with spectral reconstructions. **a**, **b** Prior EID-CT shows no clearly identifiable lesion at the affected level (arrowheads). **c**, **d** PCD-CT at 70 keV depicts a visible lesion (arrows). **e**, **f** 40-keV virtual monoenergetic image (VMI) further increases conspicuity (arrows). **g**, **h** Iodine maps show marked iodine uptake within the lesion (arrows). This example highlights improved detectability and characterization using low-keV imaging and iodine mapping

PCD-CT further enhances CNS spectral imaging via a higher spatial resolution and improved contrast-to-noise performance. Improved energy resolution enables more accurate iodine quantification and sharper delineation of tumor margins, which may be advantageous for small lesions or for tumors adjacent to bone or hemorrhage. Illustrative institutional cases suggest that 40-keV reconstructions may increase diagnostic confidence for selected intracranial lesions during routine CECT (Fig. 11) and may also provide complementary information for selected intraspinal lesions (Fig. 12). However, these observations remain preliminary and should be considered complementary to MRI rather than a substitute for dedicated neuro-oncologic MRI.

Distinguishing tumor recurrence from radiation-induced changes remains a major challenge in neuro-oncology [193–195]. Previous DECT studies, which included small cohorts, have shown that quantitative spectral parameters—including Z_eff, spectral Hounsfield unit slope, and iodine concentration—can help differentiate recurrent glioma from radiation necrosis [196]. CE-MRI and amino-acid PET remain the reference modalities. However, PCD-CT—with reduced image noise and improved material separation—may provide complementary quantitative information in selected cases where advanced imaging findings are equivocal. In addition, spectral CT plays an established role in radiation therapy planning via voxel-wise estimation of ED and stopping power ratio [197, 198]. DECT improves the accuracy of dose calculation for proton and heavy-ion therapy [199]. PCD-CT may offer additional benefits through higher spatial resolution and reduced artifacts. However, its clinical adoption for routine radiotherapy planning is still in its early phase and requires further validation—an advantage that may be particularly relevant in CNS tumors, where precise target delineation is important [200].

Overall, CNS oncology remains one of the least mature areas for spectral CT and PCD-CT evidence, and MRI remains the reference modality. Nevertheless, CNS applications represent a clinically worthwhile frontier in which PCD-CT may extend CT from morphologic assessment toward quantitative enhancement analysis, material characterization, and radiotherapy-planning support in selected problem-solving scenarios.

### Occult cancer and cancer of unknown primary (CUP)

Occult cancer and cancer of unknown primary (CUP) remain diagnostically challenging to detect because primary lesions are often small, atypically enhancing, or located in anatomically complex regions. In this context, spectral CT provides added value by improving lesion characterization and diagnostic confidence in a single whole-body examination, thereby supporting more efficient triage in occult-cancer workups [2, 201].

Although direct evidence remains limited, clinical studies have shown that spectral CT improves detection and diagnostic confidence compared with conventional CT in CUP workups, particularly in the abdominal and pelvic regions, where small, low-contrast lesions are common [1, 29, 202, 203]. Beyond lesion detection, iodine-based images and quantitative spectral parameters can identify differences in tumor vascularity and composition across primary origins [204], thereby supporting differentiation between malignant and benign lesions [100, 205] and between primary and metastatic disease [206], while providing quantitative clues to the underlying primary site. By clarifying enhancement and lesion composition at the initial CT examination, spectral CT may reduce the number of equivocal findings that prompt additional MRI, PET/CT, or short-interval follow-up. Several studies have reported significant reductions in the recommended downstream examinations for typical CUP problem sites, supporting a more streamlined and cost-conscious diagnostic pathway [2].

Spectral CT does not replace PET/CT or MRI in CUP evaluation. Nevertheless, it strengthens the initial CT assessment by improving the selection of patients who truly require advanced imaging. Evidence specific to PCD-CT in CUP remains limited. However, its improved spatial and energy resolution may further enhance the detection of subtle primary lesions and stabilize quantitative iodine assessment in routine CECT. As protocol standardization and outcome-based evidence evolve, spectral CT—and potentially PCD-CT—may assume an increasingly important role in conducting an efficient whole-body evaluation of occult cancer and CUP.

Current evidence for occult cancer/CUP applications is derived mainly from DECT studies. However, the retrospective availability of spectral reconstructions from routine PCD-CT acquisitions may make this approach especially suitable for CUP workups, where unexpectedly small or atypically enhancing lesions often require additional characterization.

## Limitations

Several practical limitations should be acknowledged. PCD-CT availability remains limited, and costs may affect adoption across institutions. Acquisition protocols, reconstruction settings, display strategies, and optimal keV selection are not yet standardized across platforms or tumor types. Quantitative spectral metrics, including iodine concentration, Z_eff, and ED, require further validation for reproducibility, particularly across vendors and detector technologies. Routine spectral reconstruction may also increase data storage requirements and interpretation burden.

At the same time, DECT literature and emerging PCD-CT evidence increasingly support the clinical utility of low-keV imaging and quantitative spectral assessment. Future studies should clarify how these advantages translate into patient outcomes, cost-effectiveness, and sustainable workflows. Although several applications discussed in this review remain perspective-oriented, they highlight promising opportunities for using PCD-CT to streamline oncologic imaging pathways and support wider clinical adoption.

## Conclusion

Spectral CT has evolved into a clinically relevant extension of routine oncologic CECT. Its most consistent contribution is improved depiction, which supports tumor detection, local extent assessment and problem-solving when conventional morphologic assessment is equivocal.

Based on current evidence, low-keV VMI, including 40-keV reconstructions when technically appropriate, may be routinely reviewed as an adjunct to conventional oncologic CECT interpretation, whereas iodine maps should be applied selectively when enhancement must be distinguished from non-enhancing tissue, necrosis, cystic components, atelectasis, or post-treatment change.

Beyond enhancement-based imaging, quantitative approaches such as Z_eff, ED, and advanced material decomposition remain investigational for most oncologic applications pending outcome-driven validation. Future work should clarify outcome-based value, protocol standardization, quantitative reproducibility, and incremental benefit over conventional CT, DECT, MRI, and PET/CT.

Overall, PCD-CT-based spectral imaging should be viewed as a practical adjunct that integrates image-quality advantages and quantitative information into oncologic CT while maintaining an evidence-informed role in clinical decision-making.

## Data Availability

The datasets generated and/or analyzed during the current study are available from the corresponding author on reasonable request. Imaging data cannot be made publicly available due to institutional restrictions and patient privacy protections.

## Abbreviations

CdTe: Cadmium telluride
CECT: Contrast-enhanced computed tomography
CE-MRI: Contrast-enhanced magnetic resonance imaging
CE-T1WI: Contrast-enhanced T1-weighted imaging
CNS: Central nervous system
CUP: Cancer of unknown primary
CZT: Cadmium zinc telluride
DECT: Dual-energy computed tomography
ED: Electron density
EID-CT: Energy-integrating detector computed tomography
HCC: Hepatocellular carcinoma
PCD-CT: Photon-counting detector computed tomography
PDAC: Pancreatic ductal adenocarcinoma
Si: Silicon
VMI: Virtual monoenergetic imaging
VNC: Virtual noncontrast
VNCa: Virtual non-calcium
Z_eff: Effective atomic number
